# The Diagnostic Performance of a Single GeneXpert MTB/RIF Assay in an Intensified Tuberculosis Case Finding Survey among HIV-Infected Prisoners in Malaysia

**DOI:** 10.1371/journal.pone.0073717

**Published:** 2013-09-09

**Authors:** Haider Abdulrazzaq Abed Al-Darraji, Humaira Abd Razak, Kee Peng Ng, Frederick L. Altice, Adeeba Kamarulzaman

**Affiliations:** 1 Centre of Excellence for Research in AIDS, Faculty of Medicine, University of Malaya, Malaysia; 2 Department of Medical Microbiology, Faculty of Medicine, University of Malaya, Malaysia; 3 AIDS Program, Department of Internal Medicine, Yale School of Medicine, New Haven, Connecticut, United States of America; 4 Division of Epidemiology of Microbial Diseases, School of Public Health, Yale University, New Haven, Connecticut, United States of America; McGill University, Canada

## Abstract

**Background:**

Delays in tuberculosis (TB) diagnosis, particularly in prisons, is associated with detrimental outcomes. The new GeneXpert MTB/RIF assay (Xpert) offers accurate and rapid diagnosis of active TB, but its performance in improving case detection in high-transmission congregate settings has yet to be evaluated. We assessed the diagnostic accuracy of a single Xpert assay in an intensified case finding survey among HIV-infected prisoners in Malaysia.

**Methods:**

HIV-infected prisoners at a single site provided two early-morning sputum specimens to be examined using fluorescence smear microscopy, BACTEC MGIT 960 liquid culture and a single Xpert. The sensitivity, specificity, negative and positive predictive values of Xpert were calculated relative to gold-standard results using MGIT 960 liquid culture. Relevant clinical and demographic data were used to examine correlates of active TB disease.

**Results:**

The majority of enrolled subjects with complete data (N=125) were men (90.4%), age <40 years (61.6%) and had injected drugs (75.2%). Median CD4 lymphocyte count was 337 cells/µL (IQR 149-492); only 19 (15.2%) were receiving antiretroviral therapy. Of 15 culture-positive TB cases, single Xpert assay accurately detected only eight previously undiagnosed TB cases, resulting in a sensitivity, specificity, positive predictive value and negative predictive value of 53.3% (95% CI 30.12-75.2%), 100% (95% CI 96.6-100%), 100% (95% CI 67.56-100%) and 94.0% (95% CI 88.2-97.1%), respectively. Only 1 of 15 (6.7%) active TB cases was smear-positive. The prevalence (12%) of undiagnosed active pulmonary TB (15 of 125 prisoners) was high and associated with longer duration of drug use (AOR 1.14, 95% CI 1.03-1.26, for each year of drug use).

**Conclusions:**

Single Xpert assay improved TB case detection and outperformed AFB smear microscopy, but yielded low screening sensitivity. Further examination of the impact of HIV infection on the diagnostic performance of the new assay alongside other screening methods in correctional settings is warranted.

## Introduction

Tuberculosis (TB) remains a major public health problem particularly in low/middle-income countries (LMIC). More than 80% of the global TB burden and TB-related deaths were reported from 22 high-burden LMIC in 2011 [1]. With the current modest annual decline in TB incidence (2%), many countries will be unable to achieve the Stop TB Partnership goal of halving TB incidence by 2015 [2]. Ambitious targets were recently set by the Partnership for the post-2015 era [3]. The spread of multidrug resistant TB (MDR-TB), the detrimental convergence with HIV infection and the unavailability of rapid diagnostic tools have contributed to the failure of global TB control [4–6]. Moreover, through altering the clinical and bacteriological presentations, HIV infection has contributed to the delay in diagnosing active TB disease, leading to increased morbidity and mortality and enhancement of transmission within communities [7–9]. These problems are further magnified in criminal justice settings where TB is a major contributing factor to the disproportionately high morbidity and mortality among prisoners [10,11]. Globally, poor prison living conditions, overcrowding, the concentration of people at high TB risk and the limited access to health services inside these congregate settings convert prisons into reservoirs fuelling the TB and MDR-TB epidemics into surrounding communities [12]. A systematic review confirmed the contribution of exposure in prisons to the population’s TB burden and showed that attributed fractions were 8.5% and 6.3% of overall TB cases in high- and low/middle-income countries, respectively [13]. Delay in TB diagnosis, due to limited screening procedures, inaccuracy of diagnostic algorithms and lack of adequate laboratory facilities, is recognized as a major limitation for TB control efforts in correctional settings [8,14–16]. This contributes not only to increased unfavorable disease outcomes but also to onward disease transmission to other inmates [9,16–20]. The current World Health Organization (WHO) symptom-based screening algorithm for prisons has proven to be diagnostically inadequate [21], therefore, in order to achieve effective TB control, the currently-recommended passive case detection needs to be coupled with an active case finding program, particularly in congregate settings and especially among HIV-infected individuals [7,22].

GeneXpert MTB/RIF (Xpert), a new nucleic acid amplification technology, offers rapid and accurate diagnostic results from biological specimens with minimal staff training requirements [20,23,24]. The analysis of this new technology as a point-of-treatment diagnostic tool in five distinct LMIC sites showed high sensitivity and specificity for the diagnosis of active TB (97% and 99.2%, respectively) and the detection of rifampicin (RIF) resistance (97.6% and 98.1%, respectively), the cornerstone of TB treatment regimens [20]. A recent review of published evaluation reports confirmed that the use of Xpert as an initial test replacing smear microscopy was highly accurate [23]. In 2010 and with the encouraging initial evaluation reports, the WHO endorsed Xpert to be used as the initial TB diagnostic test for individuals with HIV-infection or those suspected of having MDR-TB [25].

Though a dynamic transmission model predicted that the deployment of Xpert in annual TB screening surveys effectively reduces overall TB and MDR-TB prevalence in prisons of Former Soviet Union (FSU) countries, the utilization of this new technology in intensified case finding and its impact on TB burden in prisons of LMIC and where high prevalence of HIV exists has not yet been examined [26,27]. We aimed to estimate the diagnostic accuracy of a single Xpert assay and to assess correlates of active TB disease among HIV-infected inmates of a Malaysian prison.

## Methods

### Study Design

We conducted a cross-sectional intensified TB case finding survey among inmates housed in dedicated HIV units in Malaysia’s largest prison.

### Study Setting

Malaysia is a middle-income country with an estimated intermediate TB incidence and mortality rates in 2011 (81 and 5.7 per 100,000 populations, respectively). Nine percent of the overall incident TB cases were estimated to be among people living with HIV/AIDS (PLWHA) [1]. Despite the adoption of international guidelines, TB incidence in Malaysia has remained stagnant over the past ten years [1,28]. Malaysia’s incarceration rate is among the highest in the region (138 per 100,000 population), primarily attributed to the criminalization of illicit drug possession, one of the main components of the drug control policy in the country [29,30]. Moreover, people who inject drugs (PWID) represent the majority (75%) of HIV-infected individuals. HIV testing of prisoners is mandatory, with high (6%) HIV prevalence among Malaysian prisoners and 15 times higher than that of the general population [30]. HIV-infected prisoners are segregated in dedicated housing units. Prison-based TB control measures are limited and only passive case detection is deployed in prisons nationwide. The study was conducted in Kajang prison, Malaysia’s largest prison and designated as a high-security central prison located in Selangor State. Maximum prison census capacity is 3500, yet currently houses more than 4000 inmates at any one time, operating at 119% of its actual capacity [31].

### Study Population

From October to December 2012, all HIV-infected inmates housed in dedicated units in Kajang’s male and female prisons were approached for study participation. Group information sessions were conducted to assess interest and written informed consent was individually obtained thereafter. Prisoners with the following criteria were excluded from the survey: those with a negative confirmatory HIV test, those receiving anti-TB medications at the time of the survey, or those with anticipated release within 24 hours. Inmates who provided written informed consent were asked to produce two early-morning sputum specimens on two consecutive days in accordance with WHO recommendations [32]. A structured survey was used to collect socio-demographic information, incarceration history (total correctional institution entry times and years spent in incarcerations), substance abuse practices (recent heroin use within the 30 days before incarceration, any injection drug use, duration of drug use in years and current treatment with methadone within the prison) and TB and HIV clinical data (previous TB diagnosis, previous TB screening, current antiretroviral therapy prescription). For reporting TB-related symptoms, we utilized two WHO-recommended clinical algorithms: the correctional TB screening scoring and the four-symptom clinical algorithm for HIV-associated TB disease. In the former guidance, the WHO recommends screening for active TB in inmates reporting a score of 5 or more using the following scoring system: a) cough for more than 2 weeks and sputum production (2 points for each); and b) loss of weight in the past 3 months, recent loss of appetite, and chest pain (1 point for each) [22]. In the latter, the WHO recommends that the presence of any one of the following symptoms (any duration of cough, fever, night sweats and weight loss) necessitates active TB diagnostic work-up [33]. Body mass index (BMI) was calculated using weight and height and stratified as underweight if BMI <18.5 kg/m^2^. Additionally, blood samples were sent for HIV confirmation using electrochemiluminescent immunoassay technique, CD4 lymphocyte count, and quantitative HIV-1 RNA assessment.

### Sputum Specimen Collection and Processing

Enrolled subjects were asked to provide two sputum specimens (>2mL each). Sputum samples were not induced, but collection was directly-observed by one investigator (HAAA). One direct (unprocessed) sputum specimen from each participant was analyzed onsite using GeneXpert MTB/RIF v 4.3 (Cepheid, Sunnyvale, CA) according to manufacturer’s instructions using 1 mL of sputum samples and 1:2 ratio of processing solution (isopropanol and NaOH) [20]. The remainder of the first specimen and the second-day sputum specimen were sent to a quality-assured reference laboratory for smear fluorescence microscopy examination and mycobacteriological liquid culture. Upon arrival at the laboratory, samples were stored at 4 ^°^C for next-day processing, which included decontamination using N-acetyl-L-cysteine and sodium hydroxide and centrifugation before staining a sample from the deposit using Auramine-O phenol staining for fluorescence microscopy as the primary smear microscopy examination method. Regardless of smear microscopy results, pellets were inoculated into the liquid medium mycobacteria growth indicator tube (MGIT) 960 culture system (BD Diagnostics, USA). Molecular genetic Genotype Mycobacterium CM assay (Hain Lifescience, Germany) was utilized for mycobacterium species identification following positive mycobacterial growth. The reference laboratory utilizes BACTEC MGIT 960 liquid culture system to determine drug susceptibility testing (DST) for isoniazid, rifampicin, streptomycin and ethambutol with the confirmation of MDR-TB (resistance to isoniazid and rifampicin) using Genotype MTBDRplus assay (Hain Lifescience, Germany).

### Statistical Analysis

The study reporting conforms to the STARD guidelines for diagnostic accuracy reporting (www.stard-statement.org/). The collected data was entered and descriptive and logistic regression analyses were performed using SPSS v19 (IBM Corporation, Armonk, NY). Frequencies were calculated for categorical variables and median with standard deviation (SD) or interquartile range (IQR) were calculated for continuous variables depending on whether the data were normally distributed or not, respectively. Chi square and Student t-test were utilized to compare results of categorical and continuous variables between Xpert-negative/culture-positive and Xpert-positive/culture-positive active TB groups. Sensitivity, specificity, positive (PPV) and negative (NPV) predictive values of the Xpert and their respective 95% confidence intervals (95% CI) were calculated compared to the results of the gold-standard MGIT 960 liquid culture using OpenEpi v 2.3.1 [34]. . Bivariate logistic regression model was investigated and variables with significance level of p<0.2 were entered the multivariate regression analysis, and controlled for other potential confounders, to investigate correlates of active TB disease (defined by positive result on liquid culture) in this sample. Factors were considered independently associated with active TB disease at the significance level of p<0.05. Continuous variables were analyzed as numbers. The cut-off values used to dichotomize CD4 lymphocyte count (100 cells/µL) and the HIV viral load (100,000 copies/mL) were made based on the clinical significance [35]. For variables identified as being strongly collinear with each other (age, duration of drug use and duration of cigarette smoking), only one final model was used that was associated with the most optimal goodness-of-fit using the Akaike Information Criterion (AIC).

### Ethics Statement

The study protocol was reviewed and approved by the Medical Ethics Committee of the University of Malaya Medical Centre (UMMC). Following the group information session, prisoners expressing further interest in participation were met individually to be provided with explanation about the survey process and to seek written informed consent. During the confidential session, a patient information sheet that contains information about the study components and its significance to both participants and researchers was provided to each potential participant and explained thoroughly thereafter. Individuals who agreed to participate in the study were asked to sign a standard consent form, which contains details about the name, identity card number, home address and that s/he understood the nature of the study and her/his right of withdrawal at any time during the study. These data were requested in order to follow up on participants in whom TB diagnosis is made after their release. Though detailed information about the importance of early diagnosis of active TB disease among HIV-infected individuals was highlighted, we affirmed that the participation was completely voluntary. Security officers were not alerted about any Individual who chose not to participate in the survey or withdraw during the study process in order to ensure that they were not in any way disadvantaged. To further ensure this, all collected information including prisoners’ participation status was kept strictly confidential.

## Results

**Figure 1:**
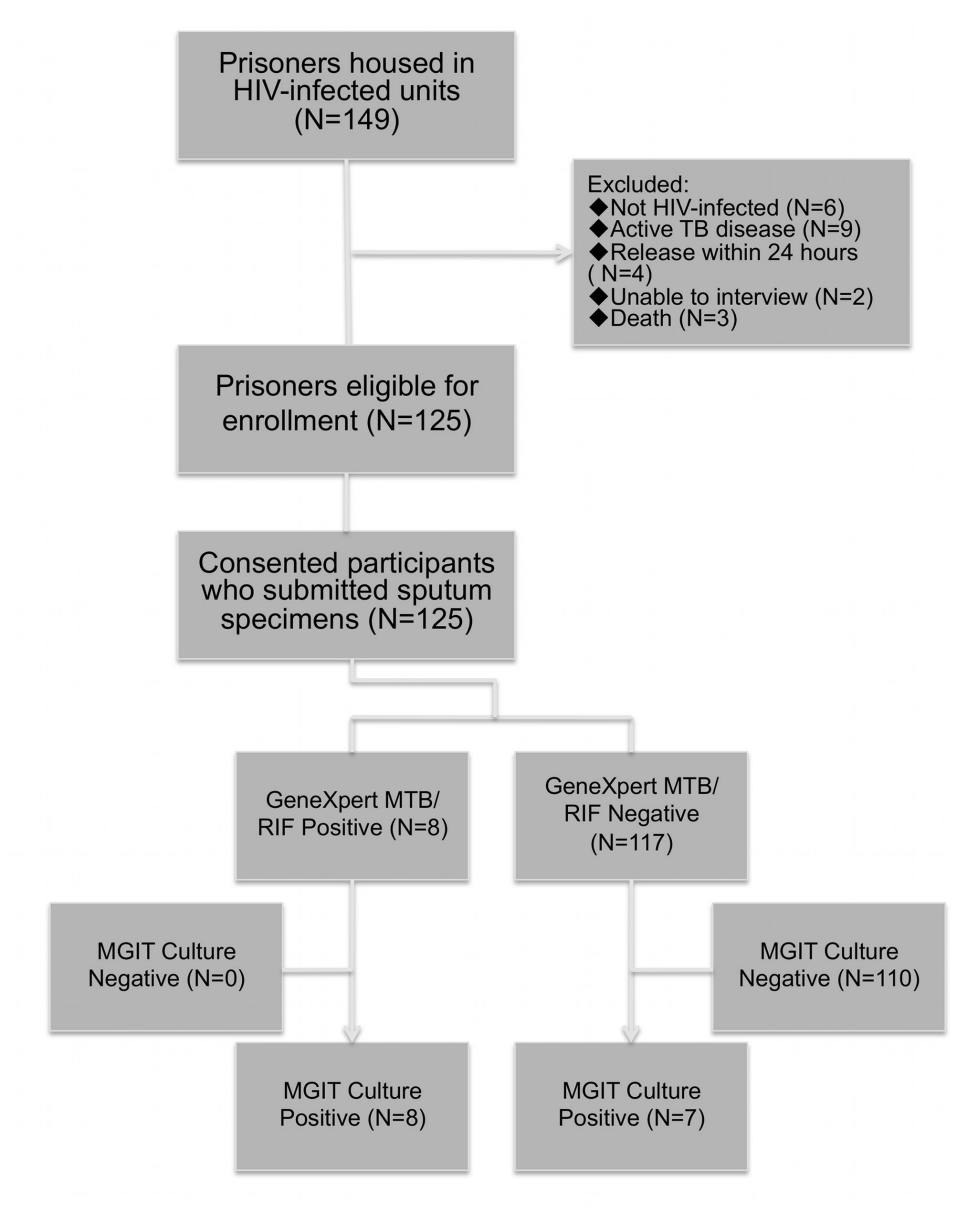
The recruitment and findings flowchart.

As of October 2012, there were 149 prisoners housed in the prison’s dedicated HIV units. Of these, 125 participants had complete data for analysis. Reasons for exclusion were (Figure 1): release within 24 hours (N=4); active TB cases currently taking anti-TB medications (N=9); considered too dangerous by prison authorities to transport (N=2); death (N=3); and negative HIV confirmatory test (N=6). Characteristics of the final sample (N=125) are summarized in Table S1.

Despite having a high risk for TB, only one-third (34.4%) of subjects reported ever being screened for TB before while 36 (28.8%) subjects reported having previously been treated for active TB, mostly (75%) within the past five years. More than half (53.6%) of those surveyed were eligible (CD4 count ≤350 cells/µL) for antiretroviral therapy (ART), but only a total of 19 (15.2%) were prescribed ART at the time of the survey. The median CD4 lymphocyte count was 337 cells/µL (IQR 149-492) and the median HIV viral load was 28,042 copies/mL (IQR 1,138-73,566.50).

Two-thirds of participants (68%) reported having at least one of the four symptoms recommended by the WHO for clinical screening algorithm among HIV-infected individuals and 10 (8%) had ≥5 points using the WHO clinical scoring system [22,33]. Neither of the two clinical screening algorithms was associated with active TB disease in this sample (OR 1.34, p=0.64 and OR 0.80, p=0.84, respectively). Median BMI was 22.3 kg/m^2^ (SD 3.25) and being underweight (BMI <18.5 Kg/m^2^) was not associated with having active TB disease (OR 1.96, p=0.43)

The study revealed a very high prevalence of undiagnosed active pulmonary TB (PTB) disease (12%, 15 out of 125 screened subjects) and overall TB prevalence when current active TB cases are added (16.7%, 24 out of 143 total HIV-infected prisoners). With the use of conventional fluorescence microscopy at the reference laboratory, all but one of the active TB cases had negative smear examination from the two sputum specimens. The single Xpert assay examination detected *Mycobacterium tuberculosis* accurately in sputa in eight (53.3%) of the culture-confirmed active TB cases. The assay however missed the diagnosis of seven active TB cases confirmed later by the liquid culture giving a sensitivity, specificity, PPV and NPV of 53.3% (95% CI 30.1-75.2%), 100% (95% CI 96.6-100%), 100% (95% CI 67.6-100%) and 94.0% (95% CI 88.2-97.1%), respectively (Table 1). Xpert was negative in a single case of *Mycobacterium* *scrofulaceum* detected subsequently by culture. Overall, the active case finding using Xpert increased TB case detection by one-fold over the current, passive, case detection (eight and nine active TB cases, respectively). Xpert-negative/ culture-positive subjects were more likely to have CD4 lymphocyte count more than 100 cells/µL (p=0.01) compared to Xpert-positive/culture-positive cases (Table 2). No rifampicin resistance was reported by Xpert in this sample, which was confirmed by the reference-laboratory mycobacteriology DST, giving 100% specificity for rifampicin resistance detection.

**Table 1:** The diagnostic accuracy of a single GeneXpert MTB/RIF assay in this sample.

Parameter	Xpert (single)	95% Confidence Interval
Sensitivity	53.3% (8 out of 15)	30.12-75.2%
Specificity	100% (110 out of 110)	96.6-100%
PPV	100% (8 out of 8)	67.56-100%
NPV	94.0% (100 out of 117)	88.2-97.1%

Xpert = GeneXpert MTB/RIF; PPV = Positive Predictive Value; NPV = Negative Predictive Value

**Table 2:** Comparative analysis between Xpert-negative/Culture-positive and Xpert-positive/Culture-positive subjects.

Variables	Categories	Xpert-/ Culture+	Xpert+/ Culture+	*P* value
Age (mean, years)		42.6	40.6	0.58
Gender	Male	7	7	0.33
	Female and transgender	0	1	
Total duration of previous incarcerations (mean, months)		83.82	66.77	0.59
Recent use of heroin	No	0	2	0.26
	Yes	7	6	
Injection drug use	No	2	1	0.44
	Yes	5	7	
Drug use duration (mean, years)		23.0	23.9	0.77
Current methadone therapy	No	7	7	0.53
	Yes	0	1	
Recent cigarette smoking	No	0	1	0.53
	Yes	7	7	
Current antiretroviral therapy	No	6	7	0.73
	Yes	1	1	
Previous history of TB	No	5	7	0.45
	Yes	2	1	
2-morning sputum specimens	No	0	1	0.53
	Yes	7	7	
WHO 4-symptom algorithm	Negative	2	2	0.66
	Positive	5	6	
WHO TB symptom scoring	<5 scores	7	7	0.53
	≥5 scores	0	1	
Body mass index (mean, Kg/m2)		21.8	20.5	0.31
CD4 lymphocyte count (cells/µL)	≤100	0	5	0.01
	>100	7	3	
HIV-1 RNA level (copies/mL)	<100,000	6	3	0.13
	**≥100,000**	**1**	**4**	

On univariate analysis (see Table S1), a number of unadjusted covariates significant at p<0.2 including age (OR 1.12, 95% CI 1.02-1.22, p=0.02), living in a crowded house before incarceration (OR 2.23, 95% CI 0.74-6.67, p=0.15), drug use duration (OR 1.14, 95% CI 1.03-1.26, p=0.01), recent alcohol use (OR 2.56, 95% CI 0.68-9.62, p=0.16), years of smoking cigarettes (OR 1.08, 95% CI 1.00-1.17, p=0.04), CD4 lymphocyte count ≤100 cells/µL (OR 3.17, 95% CI 0.95-10.55, p=0.06) and HIV-1 RNA of more than 100,000 (OR 3.04, 95% CI 0.91-10.20, p=0.07). Given that age, duration of drug use and years of cigarette smoking were identified as being collinear, separate multivariate logistic regression models using goodness-of-fit showed that the best model associated with active pulmonary TB was duration of drug use (adjusted OR 1.14, 95% CI 1.03-1.26, p=0.01 for every year of drug use). The other covariates no longer remained significant after controlling for these variables (Table S1).

## Discussion

In this intensified TB case-finding survey, we report for the first time, the diagnostic performance of a single GeneXpert MTB/RIF assay in improving pulmonary TB case detection among HIV-infected inmates of a middle-income country prison. Overall, undiagnosed active TB disease was remarkably high (12%) among this HIV-infected inmate population housed in dedicated HIV units. With a very high sputum AFB smear-negativity, a single Xpert had a limited sensitivity for the diagnosis of active TB disease (53.3%) but high specificity for active TB disease (100%) and rifampicin resistance (100%) detection compared to the liquid culture. The new convenient assay markedly increased the number of active TB cases detected in this population, potentially at much earlier TB disease burden, where profoundly symptomatic disease is common at the time of clinical presentation.

Importantly in this study, the single Xpert impressively outperformed the fluorescence smear microscopy examination. This confirms findings from earlier evaluation studies conducted in geographically distinct settings and corroborates the WHO guidance on replacing smear microscopy with Xpert as the primary diagnostic tool for active TB among HIV infected individuals [20,25,36]. Similar utilization of a single Xpert assay in an active case finding among household contacts with unknown HIV status in Tanzania had 40% higher yield than smear microscopy examination [37].

We postulated that the low sensitivity reported in this sample might be related to several factors. Cumulative data from different settings confirmed the impact of smear-negative microscopy status on the Xpert accuracy, which can be improved by increasing the number of analyzed sputa [20,23]. This observation is probably related to the low bacillary load given that Xpert requires a minimal threshold of 131 bacilli/mL (compared to 10-100 bacilli/mL for culture) [38]. Lawn SD, et al reported a similarly low (43.4%) Xpert sensitivity from a TB screening before ART initiation with prevalent smear negativity in South Africa, which improved modestly when a second sample was analyzed (62.3%) [39]. In HIV settings with considerable smear-negative TB disease, a single Xpert analysis is often deployed due to resource constraints.

HIV infection, on the other hand, complicates the diagnosis of active TB through reducing the accuracy of available diagnostic tools [40]. A diagnostic evaluation from a predominantly HIV-infected community in Tanzania showed that the vast majority (88.9%) of smear-negative TB cases were among PLWHA [41]. While HIV infection limits the sensitivity of smear microscopy in diverse settings, irrespective of the immunological status [42–44], the impact of HIV infection on Xpert diagnostic accuracy is yet to be established. In 5 LMIC sites, the overall sensitivity of Xpert was modestly lower among HIV-infected than HIV-uninfected (93.9% vs 98.4%; p=0.02) individuals [20]. A similar reduced trend in the diagnostic utility of Xpert among 496 archived sputum samples from TB suspects was found in South Africa among HIV-infected and HIV-uninfected (69.6% vs 82.9%; p=0.09) patients with significantly lower NPV (78.3% vs 90.3%; p<0.01) for HIV-infected and non-infected patients, respectively [36]. On the other hand, two clinical evaluation studies among symptomatic TB suspects in Peru [45] and Tanzania [41] reported no differences in the diagnostic accuracy of Xpert among HIV-infected and non-infected patients. A recent review of related evaluation studies revealed heterogeneity in reported accuracies of Xpert among PLWHA (70-100%) but the pooled sensitivity of Xpert was generally lower among PLWHA (76%) than HIV-uninfected subjects (89%) [23]. This heterogeneity was partially explained by the high prevalence of smear negativity among PLWHA.

Finally, intensified case finding (ICF) programs support identification of earlier TB cases, especially with less advanced TB disease and low bacterial burden, but potentially insufficient to be detected using the Xpert assay. One third of culture-confirmed active TB cases from an active pre-ART screening in South Africa were false-negative using Xpert and this phenomenon was associated with smear microscopy negativity, prolonged duration to culture positivity, less likely to have chronic cough and with less extensive radiological abnormalities [46]. Moreover, Xpert-negative active TB cases had less advanced HIV infection (higher CD4 count, lower HIV-1 RNA levels and higher BMI). Similarly, Xpert-negative cases in our report were more likely to have CD4 cell count more than 100 cells/µL, but despite having longer median time to culture positive (28 days compared to 21 days of Xpert-positive cases), the difference was not statistically significant (p=0.28). To examine this issue further, an analysis of the impact and cost of alternative diagnostic algorithms in a high TB/HIV co-infection setting projected that the addition of a second Xpert test (Xpert/Xpert) performs similarly but less expensively than the addition of culture (Xpert/culture) [47], while chest radiography performed well as a ruling-out, rather than rule-in, tool among Xpert-negative TB cases [48]. Empiric data supporting these assertions, however, are lacking.

Moreover, the finding of the low reported NVP of Xpert in this sample conforms the earlier studies of using Xpert as a rule-in rather than rule-out tool in the diagnosis of active TB among PLWHA [36]. On the other hand, Xpert was highly specific for detecting *M tuberculosis* and rifampicin resistance in this sample with no drug resistant case reported.

The deployment of Xpert in an intensified case finding program may have an immediate and sustained impact on TB epidemiology, but the finding that Xpert performed less than ideal among HIV-infected inmates suggests the need to explore combined diagnostic strategies [48–50]. In Brazil, a similar socioeconomic country, symptom-based screening was unreliable and regular mass radiographic screening was concluded to have more pronounced impact on the prevalence of active TB among prisoners [51,52]. There is a growing evidence that, whenever resources allow, bacteriological examination (smear microscopy and culture) should be provided to all PLWHA, regardless of their symptom status [53–57]. In high TB burden settings, culture yielded almost double the number of TB cases detected by smear microscopy and required fewer number needed to diagnose (NND) compare to smear microscopy (8 compared to 19). This yield, however, was more pronounced among HIV-infected compared to HIV-uninfected individuals [58].

Our survey revealed a very high prevalence (12%) of undiagnosed active TB in this prison’s HIV-infected population. Negative consequences of missing active TB cases in congregate settings are more pronounced compared to community settings [59,60]. Reports from geographically-distinct settings showed that several TB outbreaks, particularly of MDR-TB in the community were linked to exposure in prisons [61–63]. Additionally, missed infectious TB cases may undermine TB control in such high-transmission closed settings. Despite intensive TB control efforts, a single strain of *M tuberculosis* persisted in a prison in the United States and contributed to 50% of diagnosed TB cases over nine years. This was attributed to infection/re-infection and reactivation of latent TB infection (LTBI) among prisoners in contact with index cases before being diagnosed [64]. Finding that more than one third (38%) of pulmonary TB cases were missed in spite of a regular screening program in a prison in the Cameroon, Noeske et al questioned the possibility of ever achieving TB control under any conditions of confinement [60]. Moreover, missing pulmonary TB cases in prisons complicates the scaling up of two major TB control interventions in high HIV prevalence settings, namely isoniazid preventive therapy (IPT) and ART [12,17,57,65–67]. An active case finding among ambulatory PLWHA with good immunological status (CD4 count >200 cells/µL) showed that 8 of 10 PLWHA with no TB symptoms were prescribed IPT for more than 4 weeks before the bacteriological confirmation of their active TB status [10] and may potentially contribute to isoniazid resistance since a single drug was offered to active TB cases.

The overall TB prevalence (newly identified and previously diagnosed TB cases) of 16.7% is one of the highest reported prevalence of active TB from a prison setting worldwide and is 165 times higher than that in the Malaysian general population [1,22]. It is well documented that prisoners come from communities at elevated TB risk, and this may have contributed to such high TB prevalence, among others. All, but one of the study participants used illicit drugs before being incarcerated and active TB disease was independently associated with longer duration of drug use in this sample. Given this variable’s co-linearity with age, it may be that longer duration of drug use was associated with longer-standing HIV infection and increased vulnerability to TB infection. Substance misuse, particularly injection drug use, is a recognized independent risk factor for LTBI and active TB disease [68]. The high prevalence of tuberculin skin test positivity among PWID in a substance abuse treatment center (86.7%) [69] and in another Malaysian prison (87.6%) [70] confirmed the excess risk of LTBI among this population in Malaysia. Moreover, TB remains a leading cause of death among HIV infected PWID [71]. To better control co-morbid illnesses among PWID, the WHO strongly recommends the co-location of treatment for TB, HIV, and opioid dependence using a “one-stop shopping” model of care, but many countries facing the detrimental convergence of these epidemics run these programs vertically [71–73]. The availability and impact of utilizing new rapid diagnostic tools, like the one used in this study, improve the likelihood of better integration of services to treat multiple co-morbidities, yet needs further empiric investigation.

Prisons have been identified as reservoirs for TB, ultimately spreading the disease to the general population through prison staff and released inmates. Increasing incarceration rates alone was projected to increase TB and MDR-TB incidence in civilian communities of the FSU [22,74]. Finding alternatives to incarceration through penal reforms, particularly for PWID, in an attempt to reduce overcrowding would likely reduce TB burden in similar communities [74]. Proposed alternatives include drug courts with alternatives to incarceration, noncustodial measures, enhanced bail procedures and adapted sentence policies [27]. In a similar setting, the diversion of PWID from prisons into community-based treatment facilities was a potential contributory factor leading to the reported reduction in TB prevalence in 27 Thai prisons over a six-year period [75].

### Study limitations

Though the findings are compelling for the use of Xpert in TB case finding among HIV-infected prisoners, this study is limited by its small sample size. The cross-sectional survey is limited in that it can only demonstrate associations and not causality, but nonetheless, it was implemented, in a manner used by prison-based screening efforts elsewhere to screen for active TB. Moreover, the sample is entirely representative since all HIV-infected prisoners in Malaysia’s largest prison were sampled, including a high acceptance rate. Also, the lack of available chest radiography limited our ability to use all TB diagnostic modalities. Last, only a single Xpert assay was deployed, perhaps reducing sensitivity, yet prisons experience considerable budgetary constraints and this approach likely represents real-world prison-based screening methods.

## Conclusions

In this high HIV and TB prevalence setting, active case finding using a single Xpert analysis has increased active pulmonary TB case detection among HIV-infected Malaysian prisoners. The new diagnostic tool is markedly superior to sputum smear microscopy, however, missed almost half of previously undiagnosed pulmonary TB cases (sensitivity of 53.3%) in the predominantly smear-negative sample. The extraordinarily high TB prevalence reported here affirms the importance of intensified TB case finding in congregate settings and warrants further exploration of multiple diagnostic approaches using the Xpert technology, potentially combined with other diagnostic screening modalities in correctional institutions with high HIV prevalence.

## Supporting Information

Table S1 **Correlates of Undiagnosed Active Pulmonary Tuberculosis.**
(DOCX)

## References

[1] World Health Organization (WHO) (2012)Global Tuberculosis Report 2012 Geneva: Elsevier.

[2] Lönnroth K, Corbett E, Golub J, Uplekar M, Weil D et al. (2013) Systematic screening for active tuberculosis : rationale, definitions and key considerations. Int J Tuberc Lung Dis 17: 289–298. doi:10.5588/ijtld.12.0797. PubMed: 23407219. 23407219 10.5588/ijtld.12.0797

[3] Maxmen A (2013) Ahead of WHO meeting, experts clash over tuberculosis targets. Nat Med 19: 115. doi:10.1038/nm0213-115. PubMed: 23389590. 23389590 10.1038/nm0213-115

[4] Lawn SD, Zumla AI (2011) Tuberculosis. Lancet 378: 57–72. doi:10.1016/S0140-6736(10)62173-3. PubMed: 21420161. 21420161 10.1016/S0140-6736(10)62173-3

[5] Zumla A, Raviglione M, Hafner R, Von Reyn CF (2013) Tuberculosis. N Engl J Med 368: 745–755. doi:10.1056/NEJMra1200894. PubMed: 23425167. 23425167 10.1056/NEJMra1200894

[6] Corbett EL, Marston B, Churchyard GJ, De Cock KM (2006) Tuberculosis in sub-Saharan Africa : opportunities, challenges, and change in the era of antiretroviral treatment. Lancet 367: 926–937. doi:10.1016/S0140-6736(06)68383-9. PubMed: 16546541. 16546541 10.1016/S0140-6736(06)68383-9

[7] Golub JE, Mohan CI, Comstock GW, Chaisson RE (2005) Active case finding of tuberculosis: historical perspective and future prospects. Int J Tuberc Lung Dis 9: 1183–1203. PubMed: 16333924. 16333924 PMC4472641

[8] Wood R, Middelkoop K, Myer L, Grant AD, Whitelaw A et al. (2007) Undiagnosed tuberculosis in a community with high HIV prevalence: implications for tuberculosis control. Am J Respir Crit Care Med 175: 87–93. doi:10.1164/rccm.200606-759OC. PubMed: 16973982. 16973982 10.1164/rccm.200606-759OC PMC1899262

[9] Davis JL, Worodria W, Kisembo H, Metcalfe JZ, Cattamanchi A et al. (2010) Clinical and radiographic factors do not accurately diagnose smear-negative tuberculosis in HIV-infected inpatients in Uganda: a cross-sectional study. PLOS ONE 5: e9859. doi:10.1371/journal.pone.0009859. PubMed: 20361038. 20361038 10.1371/journal.pone.0009859 PMC2845634

[10] Mtei L, Matee M, Herfort O, Bakari M, Horsburgh CR et al. (2005) High rates of clinical and subclinical tuberculosis among HIV-infected ambulatory subjects in Tanzania. Clin Infect Dis 40: 1500–1507. doi:10.1086/429825. PubMed: 15844073. 15844073 10.1086/429825

[11] O’Grady J, Hoelscher M, Atun R, Bates M, Mwaba P et al. (2011) Tuberculosis in prisons in sub-Saharan Africa--the need for improved health services, surveillance and control. Tuberculosis (Edinb, Scotland) 91: 173–178. doi:10.1016/j.tube.2010.12.002. 10.1016/j.tube.2010.12.002 21251881

[12] Getahun H, Gunneberg C, Sculier D, Verster A, Raviglione M (2012) Tuberculosis and HIV in people who inject drugs: evidence for action for tuberculosis, HIV, prison and harm reduction services. Current Opinion HIV Aids 7: 345–353. doi:10.1097/COH.0b013e328354bd44. PubMed: 22678489. 10.1097/COH.0b013e328354bd44 22678489

[13] Baussano I, Williams BG, Nunn P, Beggiato M, Fedeli U et al. (2010) Tuberculosis incidence in prisons: a systematic review. PLOS Med 7: e1000381 PubMed: 21203587. 21203587 10.1371/journal.pmed.1000381 PMC3006353

[14] Vinkeles Melchers NVS, Van Elsland SL, Lange JMa, Borgdorff MW, Van den Hombergh J (2013) State of Affairs of Tuberculosis in Prison Facilities: A Systematic Review of Screening Practices and Recommendations for Best TB Control. PLOS ONE 8: e53644. doi:10.1371/journal.pone.0053644. PubMed: 23372662. 23372662 10.1371/journal.pone.0053644 PMC3556085

[15] Saunders DL, Olive DM, Wallace SB, Lacy D, Leyba R et al. (2001) Tuberculosis screening in the federal prison system: an opportunity to treat and prevent tuberculosis in foreign-born populations. Public Health Rep (Washington, DC: 1974 116: 210–218. PubMed: 12034910. 10.1016/S0033-3549(04)50036-5 PMC1497313 12034910

[16] Reid SE, Topp SM, Turnbull ER, Hatwiinda S, Harris JB et al. (2012) Tuberculosis and HIV control in sub-Saharan African prisons: “thinking outside the prison cell”. J Infect Dis 205 Suppl: S265–S273. doi:10.1093/infdis/jis029. PubMed: 22448015. 22448015 10.1093/infdis/jis029

[17] Getahun H, Kittikraisak W, Heilig CM, Corbett EL, Ayles H et al. (2011) Development of a standardized screening rule for tuberculosis in people living with HIV in resource-constrained settings: individual participant data meta-analysis of observational studies. PLOS Med 8: e1000391. 21267059 10.1371/journal.pmed.1000391 PMC3022524

[18] Kranzer K, Houben RM, Glynn JR, Bekker L-G, Wood R et al. (2010) Yield of HIV-associated tuberculosis during intensified case finding in resource-limited settings: a systematic review and meta-analysis. Lancet Infect Dis 10: 93–102. doi:10.1016/S1473-3099(09)70326-3. PubMed: 20113978. 20113978 10.1016/S1473-3099(09)70326-3 PMC3136203

[19] Alvarez-Uria G, Azcona JM, Midde M, Naik PK, Reddy S et al. (2012) Rapid Diagnosis of Pulmonary and Extrapulmonary Tuberculosis in HIV-Infected Patients. Comparison of LED Fluorescent Microscopy and the GeneXpert MTB/RIF Assay in a District Hospital in India. Tuberculosis Res Treat: 2012: Article ID 932862, 4 pages 10.1155/2012/932862 PMC3433122 22966426

[20] Boehme CC, Nabeta P, Hillemann D, Nicol MP, Shenai S et al. (2010) Rapid Molecular Detection of Tuberculosis and Rifampin Resistance. N Engl J Med 363: 1005–1015. doi:10.1056/NEJMoa0907847. PubMed: 20825313. 20825313 10.1056/NEJMoa0907847 PMC2947799

[21] Fournet N, Sanchez A, Massari V, Penna L, Natal S et al. (2006) Development and evaluation of tuberculosis screening scores in Brazilian prisons. Public Health 120: 976–983. doi:10.1016/j.puhe.2006.06.004. PubMed: 16965796. 16965796 10.1016/j.puhe.2006.06.004

[22] World Health Organization (WHO) (2000) Tuberculosis Control in Prisons: A Manual for Programme Managers. Geneva.

[23] Steingart K, Sohn H, Schiller I, Kloda L, Boehme C et al. (2013) Xpert® MTB / RIF assay for pulmonary tuberculosis and rifampicin resistance in adults (Review ). Cochrane Database of Systematic Reviews: Art. No: CD009593 10.1002/14651858.CD009593.pub2 PMC4470352 23440842

[24] Chang K, Lu W, Wang J, Zhang K, Jia S et al. (2012) Rapid and effective diagnosis of tuberculosis and rifampicin resistance with Xpert MTB/RIF assay: a meta-analysis. J Infect 64: 580–588. doi:10.1016/j.jinf.2012.02.012. PubMed: 22381459. 22381459 10.1016/j.jinf.2012.02.012

[25] World Health Organization (WHO) (2011) Rapid Implementation of the Xpert MTB / RIF diagnostic test: technical and operational “How-to”; practical considerations. Geneva.

[26] Winetsky DE, Negoescu DM, DeMarchis EH, Almukhamedova O, Dooronbekova A et al. (2012) Screening and rapid molecular diagnosis of tuberculosis in prisons in Russia and Eastern Europe: a cost-effectiveness analysis. PLOS Med 9: e1001348 PubMed: 23209384. 23209384 10.1371/journal.pmed.1001348 PMC3507963

[27] Reid SE, Topp SM, Turnbull ER, Hatwiinda S, Harris JB et al. (2012) Tuberculosis and HIV control in sub-Saharan African prisons: “thinking outside the prison cell”. J Infect Dis 205 Suppl: S265–S273. doi:10.1093/infdis/jis029. PubMed: 22448015. 22448015 10.1093/infdis/jis029

[28] Iyawoo K (2004) Tuberculosis in Malaysia: problems and prospect of treatment and control. Tuberculosis 84: 4–7. doi:10.1016/j.tube.2003.08.014. PubMed: 14670340. 14670340 10.1016/j.tube.2003.08.014

[29] Walmsley R (2011) World Prison Population List (ninth edition).

[30] Choi P, Kavasery R, Desai MM, Govindasamy S, Kamarulzaman A et al. (2010) Prevalence and correlates of community re-entry challenges faced by HIV-infected male prisoners in Malaysia. Int J STD AIDS 21: 416–423. doi:10.1258/ijsa.2009.009180. PubMed: 20606222. 20606222 10.1258/ijsa.2009.009180 PMC2925151

[31] SURUHANJAYA HAK ASASI MANUSIA MALAYSIA (2008). The State Prisons Immigration Detention Cent Malays: 2007-2008. Available: http://www.suhakam.org.my/c/document_library/get_file?p_l_id=10408&folderId=236834&name=DLFE-7802.pdf.

[32] World Health Organization (WHO) (2006) Improving the diagnosis and treatment of smear-negative pulmonary and extrapulmonary tuberculosis among adults and adolescents. Recommendations for HIV-prevalent and resource-constrained settings. Geneva.

[33] World Health Organization (WHO) (2011) Guidelines for intensified tuberculosis case-finding and isoniazid preventive therapy for people living with HIV in resource- constrained settings. Geneva.

[34] Dean AG, Sullivan KM, Soe MM (2011) OpenEpi: Open Source Epidemiologic Statistics for Public Health. Available: www.OpenEpi.com. Accessed 3 March 2013.

[35] Kufa T, Mngomezulu V, Charalambous S, Hanifa Y, Fielding K et al. (2012) Undiagnosed tuberculosis among HIV clinic attendees: association with antiretroviral therapy and implications for intensified case finding, isoniazid preventive therapy, and infection control. J Acquir Immune Defic Syndr, 60: e22–8 (1999) 60: e22– 8. PubMed: 22627184 10.1097/QAI.0b013e318251ae0b 22627184

[36] Theron G, Peter J, Van Zyl-Smit R, Mishra H, Streicher E et al. (2011) Evaluation of the Xpert MTB/RIF assay for the diagnosis of pulmonary tuberculosis in a high HIV prevalence setting. Am J Respir Crit Care Med 184: 132–140. doi:10.1164/rccm.201101-0056OC. PubMed: 21493734. 21493734 10.1164/rccm.201101-0056OC

[37] Ntinginya EN, Squire SB, Millington Ka, Mtafya B, Saathoff E et al. (2012) Performance of the Xpert® MTB/RIF assay in an active case-finding strategy: a pilot study from Tanzania. Int J Tuberc Lung Dis 16: 1468–1470. doi:10.5588/ijtld.12.0127. PubMed: 22964006. 22964006 10.5588/ijtld.12.0127

[38] Kirwan DE, Cárdenas MK, Gilman RH (2012) Rapid implementation of new TB diagnostic tests: is it too soon for a global roll-out of Xpert MTB/RIF? Am J Trop Med Hyg 87: 197–201. doi:10.4269/ajtmh.2012.12-0107. PubMed: 22855746. 22855746 10.4269/ajtmh.2012.12-0107 PMC3414551

[39] Lawn SD, Brooks SV, Kranzer K, Nicol MP, Whitelaw A et al. (2011) Screening for HIV-associated tuberculosis and rifampicin resistance before antiretroviral therapy using the Xpert MTB/RIF assay: a prospective study. PLOS Med 8: e1001067 PubMed: 21818180. 21818180 10.1371/journal.pmed.1001067 PMC3144215

[40] Laserson KF, Wells CD (2007) Reaching the targets for tuberculosis control: the impact of HIV. Bull World Health Organ 85: 377–381. doi:10.2471/BLT.06.035329. PubMed: 17639223. 17639223 10.2471/06-035329 PMC2636651

[41] Rachow A, Zumla A, Heinrich N, Rojas-Ponce G, Mtafya B et al. (2011) Rapid and accurate detection of Mycobacterium tuberculosis in sputum samples by Cepheid Xpert MTB/RIF assay-a clinical validation study. PLOS ONE 6: e20458. doi:10.1371/journal.pone.0020458. PubMed: 21738575. 21738575 10.1371/journal.pone.0020458 PMC3126807

[42] Cattamanchi A, Dowdy DW, Davis JL, Worodria W, Yoo S et al. (2009) Sensitivity of direct versus concentrated sputum smear microscopy in HIV-infected patients suspected of having pulmonary tuberculosis. BMC Infect Dis 9: 53. doi:10.1186/1471-2334-9-53. PubMed: 19419537. 19419537 10.1186/1471-2334-9-53 PMC2690598

[43] Swaminathan S, Padmapriyadarsini C, Narendran G (2010) HIV-associated tuberculosis: clinical update. Clin Infect Dis 50: 1377–1386. doi:10.1086/652147. PubMed: 20388036. 20388036 10.1086/652147

[44] Gupta RK, Lawn SD, Bekker LG, Caldwell J, Kaplan R et al. (2013) Impact of human immunodeficiency virus and CD4 count on tuberculosis diagnosis : analysis of city-wide data from Cape Town, South Africa. Int J Tuberc Lung Dis 17: 1014–1022. doi:10.5588/ijtld.13.0032. PubMed: 23827024. 23827024 10.5588/ijtld.13.0032 PMC3990260

[45] Carriquiry G, Otero L, González-Lagos E, Zamudio C, Sánchez E et al. (2012) A diagnostic accuracy study of Xpert®MTB/RIF in HIV-positive patients with high clinical suspicion of pulmonary tuberculosis in Lima, Peru. PLOS ONE 7: e44626. doi:10.1371/journal.pone.0044626. PubMed: 22970271. 22970271 10.1371/journal.pone.0044626 PMC3436871

[46] Lawn SD, Kerkhoff AD, Vogt M, Ghebrekristos Y, Whitelaw A et al. (2012) Characteristics and early outcomes of patients with Xpert MTB/RIF-negative pulmonary tuberculosis diagnosed during screening before antiretroviral therapy. Clin Infect Dis 54: 1071–1079. doi:10.1093/cid/cir1039. PubMed: 22318975. 22318975 10.1093/cid/cir1039 PMC3309885

[47] Schnippel K, Meyer-Rath G, Long L, Stevens WS, Sanne I et al. (2014) Diagnosing Xpert MTB / RIF-negative TB : Impact and cost of alternative algorithms for South Africa. S Afr Med J 103: 101–106. 10.7196/samj.6182 23374320

[48] Theron G, Pooran A, Peter J, Van Zyl-Smit R, Kumar Mishra H et al. (2012) Do adjunct tuberculosis tests, when combined with Xpert MTB/RIF, improve accuracy and the cost of diagnosis in a resource-poor setting? Eur Respir J 40: 161–168. doi:10.1183/09031936.00145511. PubMed: 22075479. 22075479 10.1183/09031936.00145511 PMC5523948

[49] Menzies Na, Cohen T, Lin H-H, Murray M, Salomon J a (2012) Population health impact and cost-effectiveness of tuberculosis diagnosis with Xpert MTB/RIF: a dynamic simulation and economic evaluation. PLOS Med 9: e1001347 PubMed: 23185139. 23185139 10.1371/journal.pmed.1001347 PMC3502465

[50] Lin H-H, Dowdy D, Dye C, Murray M, Cohen T (2012) The impact of new tuberculosis diagnostics on transmission: why context matters. Bull World Health Organ 90: 739–747A. doi:10.2471/BLT.11.101436. PubMed: 23109741. 23109741 10.2471/BLT.11.101436 PMC3471051

[51] Sanchez a, Gerhardt G, Natal S, Capone D, Espinola A, et al (2005) Prevalence of pulmonary tuberculosis and comparative evaluation of screening strategies in a Brazilian prison. Int J Tuberc Lung Dis 9: 633–639. PubMed: 15971390. 15971390

[52] Legrand J, Sanchez A, Le Pont F, Camacho L, Larouze B (2008) Modeling the impact of tuberculosis control strategies in highly endemic overcrowded prisons. PLOS ONE 3: e2100. doi:10.1371/journal.pone.0002100. PubMed: 18461123. 18461123 10.1371/journal.pone.0002100 PMC2324198

[53] Lawn SD, Harries AD, Meintjes G, Getahun H, Havlir DV et al. (2012) Reducing deaths from tuberculosis in antiretroviral treatment programmes in sub-Saharan Africa. AIDS Lond Engl 26: 2121–2133. doi:10.1097/QAD.0b013e3283565dd1. PubMed: 22695302. 10.1097/QAD.0b013e3283565dd1 PMC3819503 22695302

[54] Ngadaya ES, Mfinanga GS, Wandwalo ER, Morkve O (2009) Detection of pulmonary tuberculosis among patients with cough attending outpatient departments in Dar Es Salaam, Tanzania: does duration of cough matter? BMC Health Serv Res 9: 112. doi:10.1186/1472-6963-9-112. PubMed: 19570233. 19570233 10.1186/1472-6963-9-112 PMC2713219

[55] Nguyen DTM, Hung NQ, Giang LT, Dung NH, Lan NTN et al. (2011) Improving the diagnosis of pulmonary tuberculosis in HIV-infected individuals in Ho Chi Minh City, Viet Nam. Int J Tuberc Lung Dis 15: 1528–1534. doi:10.5588/ijtld.10.0777. PubMed: 22008768. 22008768 10.5588/ijtld.10.0777

[56] Oni T, Burke R, Tsekela R, Bangani N, Seldon R et al. (2011) High prevalence of subclinical tuberculosis in HIV-1-infected persons without advanced immunodeficiency: implications for TB screening. Thorax 66: 669–673. doi:10.1136/thx.2011.160168. PubMed: 21632522. 21632522 10.1136/thx.2011.160168 PMC3142344

[57] Rangaka MX, Wilkinson RJ, Glynn JR, Boulle A, Van Cutsem G et al. (2012) Effect of antiretroviral therapy on the diagnostic accuracy of symptom screening for intensified tuberculosis case finding in a South African HIV clinic. Clin Infect Dis 55: 1698–1706. doi:10.1093/cid/cis775. PubMed: 22955441. 22955441 10.1093/cid/cis775 PMC3501332

[58] Demers A-M, Verver S, Boulle A, Warren R, Van Helden P et al. (2012) High yield of culture-based diagnosis in a TB-endemic setting. BMC Infect Dis 12: 218. doi:10.1186/1471-2334-12-218. PubMed: 22978323. 22978323 10.1186/1471-2334-12-218 PMC3482573

[59] Stead WW (1978) Undetected tuberculosis in prison. Source of infection for community at large. JAMA 240: 2544–2547. doi:10.1001/jama.240.23.2544. PubMed: 712956. 712956 10.1001/jama.240.23.2544

[60] Noeske J, Ndi N, Mbondi S (2011) Controlling tuberculosis in prisons against confinement conditions: a lost case? Experience Cameroon Int J Tuberc Lung Dis 15: 223–227. 21219685

[61] Fernandez de la Hoz K, Iñigo J, Fernandez-Martín JI, Arce A, Alonso-Sanz M et al. (2001) The influence of HIV infection and imprisonment on dissemination of Mycobacterium tuberculosis in a large Spanish city. Int J Tuberc Lung Dis 5: 696–702. PubMed: 11495258. 11495258

[62] Sosa LE, Lobato MN, Condren T, Williams MN, Hadler JL (2008) Outbreak of tuberculosis in a correctional facility. Int J Tuberc Lung Dis 12: 689–691. PubMed: 18492339. 18492339

[63] Portugal I, Covas MJ, Brum L, Viveiros M, Ferrinho P et al. (1999) Outbreak of multiple drug-resistant tuberculosis in Lisbon : detection by restriction fragment length polymorphism analysis. Int J Tuberc Lung Dis 3: 207–213. PubMed: 10094321. 10094321

[64] Ijaz K, Yang Z, Templeton G, Stead WW, Bates JH et al. (2004) Persistence of a strain of Mycobacterium tuberculosis in a prison system. Int J Tuberc Lung Dis 8: 994–1000. PubMed: 15305483. 15305483

[65] Van Rie A, Page-Shipp L, Scott L, Sanne I, Stevens W (2010) Xpert(®) MTB/RIF for point-of-care diagnosis of TB in high-HIV burden, resource-limited countries: hype or hope? Expert Rev Mol Diagn 10: 937–946. doi:10.1586/erm.10.67. PubMed: 20964612. 20964612 10.1586/erm.10.67

[66] Reid MJa, Shah NS (2009) Approaches to tuberculosis screening and diagnosis in people with HIV in resource-limited settings. Lancet Infect Dis 9: 173–184. doi:10.1016/S1473-3099(09)70043-X. PubMed: 19246021. 19246021 10.1016/S1473-3099(09)70043-X

[67] Al-Darraji HAA, Kamarulzaman A, Altice FL (2012) Isoniazid preventive therapy in correctional facilities : a systematic review. Int J Tuberc Lung Dis 16: 871–879. doi:10.5588/ijtld.11.0447. PubMed: 22410101. 22410101 10.5588/ijtld.11.0447

[68] Deiss RG, Rodwell TC, Garfein RS (2009) Tuberculosis and illicit drug use: review and update. Clin Infect Dis 48: 72–82. doi:10.1086/594126. PubMed: 19046064. 19046064 10.1086/594126 PMC3110742

[69] Wong K, Gan D, Al-Darraji H, Fu J, Loeliger K et al. (2012) High prevalence of latent tuberculosis infection among attendees of a Malaysian Drug Rehabilitation Centre (Oral Presentation). 43rd Union World Conference on Lung Health, Kuala Lumpur.

[70] Margolis B, Al-Darraji HAA, Wickersham JA, Kamarulzaman A, Altice FL (2013) Prevalence of Tuberculosis Symptoms and Latent Tuberculosis Infection among Prisoners in Northeastern Malaysia. Int J Tuberc Lung Dis (In press). 10.5588/ijtld.13.0193 PMC3913085 24200265

[71] Sylla L, Bruce RD, Kamarulzaman A, Altice FL (2007) Integration and co-location of HIV/AIDS, tuberculosis and drug treatment services. Int J Drug Policy 18: 306–312. doi:10.1016/j.drugpo.2007.03.001. PubMed: 17689379. 17689379 10.1016/j.drugpo.2007.03.001 PMC2696234

[72] World Health Organization (WHO) (2008) Policy Guidelines for Collaborative TB and HIV Services for Injecting and Other Drug Users: An Integrated Approach. Geneva. 26447262

[73] Fu JJ, Bazazi AR, Altice FL, Mohamed MN, Kamarulzaman A (2012) Absence of antiretroviral therapy and other risk factors for morbidity and mortality in Malaysian compulsory drug detention and rehabilitation centers. PLOS ONE 7: e44249. doi:10.1371/journal.pone.0044249. PubMed: 23028508. 23028508 10.1371/journal.pone.0044249 PMC3445567

[74] Stuckler D, Basu S, McKee M, King L (2008) Mass incarceration can explain population increases in TB and multidrug-resistant TB in European and central Asian countries. Proc Natl Acad Sci U S A 105: 13280–13285. doi:10.1073/pnas.0801200105. PubMed: 18728189. 18728189 10.1073/pnas.0801200105 PMC2533181

[75] Jittimanee SX, Ngamtrairai N, White MC, Jittimanee S (2007) A prevalence survey for smear-positive tuberculosis in Thai prisons. Int J Tuberc Lung Dis 11: 556–561. PubMed: 17439681. 17439681

