# Supplementary material for: The Diagnostic Performance of a Single GeneXpert MTB/RIF Assay in an Intensified Tuberculosis Case Finding Survey among HIV-Infected Prisoners in Malaysia
Source: PLoS One. 2013 Sep 9;8(9):e73717. doi: 10.1371/journal.pone.0073717 (PMC3767617; doi:10.1371/journal.pone.0073717)
Supplement: Table S1 — Correlates of Undiagnosed Active Pulmonary Tuberculosis. (DOCX) [file pone.0073717.s001.docx]

Table S1: Correlates of Undiagnosed Active Pulmonary Tuberculosis

| **Covariates** | Categories | N=125 | Univariate Analysis | | Multivariate Analysis | |
| --- | --- | --- | --- | --- | --- | --- |
|  |  |  | Unadjusted OR (95% CI) | P-value | Adjusted OR (95% CI) | P-value |
| **Median age, year (S.D.)** |  | 37.0 (SD 6.6) | 1.12 (1.02-1.22) | **0.02** | ** |  |
| **Sex** | Female or transgender | 12 (9.6%) | Referent |  |  |  |
|  | Male | 113 (90.4%) | 1.55 (0.18-12.98) | 0.68 |  |  |
| **Completed primary education** | Yes | 108 (86.4%) | Referent |  |  |  |
|  | No | 17 (13.6%) | 0.42 (0.05-3.42) | 0.42 |  |  |
| **Pre-incarceration crowded housing** | No | 87 (69.6%) | Referent |  |  |  |
|  | Yes | 38 (30.4%) | 2.23 (0.74-6.67) | **0.15** | 2.38 (0.67-8.40) | 0.17 |
| **Median total duration of previous incarceration, months (IQR)** |  | 66.70 (27.33-121.48) | 1 (0.99-1.01) | 0.62 |  |  |
| **Median duration of drug use, years (S.D.)** |  | 20.1 (6.8) | 1.14 (1.03-1.26) | **0.01** | 1.14 (1.03-1.26) | 0.01 |
| **Recent alcohol use*** | No | 79 (63.2%) | Referent |  |  |  |
|  | Yes | 46 (36.8%) | 2.56 (0.68-9.62) | **0.16** | 0.54 (0.12-2.37) | 0.41 |
| **Recent heroin use*** | No | 29 (23.2%) | Referent |  |  |  |
|  | Yes | 96 (76.8%) | 2.11 (0.45-9.97) | 0.34 |  |  |
| **Injection drug use, ever** | No | 31 (24.8%) | Referent |  |  |  |
|  | Yes | 94 (75.2%) | 1.37 (0.36-5.20) | 0.65 |  |  |
| **Receiving methadone maintenance therapy** | No | 106 (84.8%) | Referent |  |  |  |
|  | Yes | 19 (15.2%) | 2.74 (0.34-22.16) | 0.34 |  |  |
| **Recent cigarette smoking*** | No | 8 (6.4%) | Referent |  |  |  |
|  | Yes | 117 (93.6%) | 0.95 (0.11-8.32) | 0.96 |  |  |
| **Median cigarette smoking duration, years (S.D.)** |  | 23.2 (7.0) | 1.08 (1.00-1.17) | **0.04** | ** |  |
| **Receiving antiretroviral therapy** | Yes | 19 (15.2%) | Referent |  |  |  |
|  | No | 106 (84.8%) | 1.18 (0.25-5.74) | 0.83 |  |  |
| **Previously treated for tuberculosis** | No | 89 (71.2%) | Referent |  |  |  |
|  | Yes | 36 (28.8%) | 0.58 (0.15-2.20) | 0.43 |  |  |
| **Cough ≥ 2weeks** | No | 111 (88.8%) | Referent |  |  |  |
|  | Yes | 14 (11.2%) | 0.53 (0.06-4.40) | 0.56 |  |  |
| **WHO 4-symptom algorithm screen** | Negative | 40 (32.0%) | Referent |  |  |  |
|  | Positive | 85 (68.0%) | 1.34 (0.39-4.49) | 0.64 |  |  |
| **WHO Prison Tuberculosis Scoring Algorithm** | <5 points | 115 (92.0%) | Referent |  |  |  |
|  | ≥5 points | 10 (8.0%) | 0.80 (0.09-6.81) | 0.84 |  |  |
| **Body mass index (kg/m^2^)** | ≥18.5 | 116 (92.8%) | Referent |  |  |  |
|  | <18.5 | 9 (7.2%) | 1.96 (0.37-10.25) | 0.43 |  |  |
| **CD4 lymphocyte count (cells/mL)** | ≤100 | 105 (84.0%) | Referent |  |  |  |
|  | >100 | 20 (16.0%) | 3.17 (0.95-10.55) | **0.06** | 1.69 (0.39-7.54) | 0.49 |
| **HIV-1 RNA level (copies/mL)** | <100,000 | 102 (81.6%) | Referent |  |  |  |
|  | ≥100,000 | 22 (17.6%) | 3.04 (0.91-10.20) | **0.07** | 1.62 (0.36-7.34) | 0.53 |
| **AIC** |  |  |  |  | 81.05 |  |

**Legend**: S.D.=standard deviation; IQR= Interquartile range; WHO=World Health Organization; AIC=Akaike Information Criterion; *Recent refers to the 30 days before incarceration; ** Continuous variables collinear with duration of drug use.
